# A nomogram for predicting early thrombolytic efficacy in stroke patients

**DOI:** 10.3389/fneur.2025.1452856

**Published:** 2025-08-20

**Authors:** Chen Lou, Dongjuan Xu

**Affiliations:** Department of Neurology, Dongyang People’s Hospital, Affiliated to Wenzhou Medical University, Dongyang, China

**Keywords:** cerebral infarction, ischemic stroke, intravenous thrombolysis, nomogram, early thrombolytic efficacy

## Abstract

**Background:**

The purpose of this study was to develop and verify a novel nomogram for predicting stroke patients’ early thrombolytic efficacy.

**Methods:**

We collect basic facts and clinical data of stroke patients with intravenous thrombolysis. A nomogram was established for predicting early thrombolytic efficacy in these people. The LASSO regression method and multivariate logistic regression were used to filter variables and choose predictors. Predictors were applied to develop a model. The model’s discriminatory capacity was assessed by computing the area under the curve. In addition, the model’s calibration analysis and decision curve analysis were performed.

**Results:**

Using multivariate logistic regression and LASSO regression techniques, five variables were chosen. These variables were age, NIHSS score before thrombolysis, prothrombin time, neutrophil count, and monocyte count. The AUC of the prediction model was 0.761 (95% CI, 0.717–0.805) in the training set and 0.744 (95% CI, 0.653–0.835) in the test set. The decision curve showed that the threshold probabilities for the effectiveness of early thrombolysis in cerebral infarction are 25–67% and 25–73% in the training set and test set, respectively.

**Conclusion:**

A novel nomogram with age, NIHSS score before thrombolysis, prothrombin time, neutrophil count, and monocyte count as variables has the potential to predict early thrombolytic efficacy in stroke patients. Physicians could utilize this handy nomogram to make better decisions for stroke patients with intravenous thrombolysis.

## 1 Introduction

Stroke is a common and frequent neurological disorder. It can result in an extensive range of neurological impairments such as communication, walking, swallowing, and other abilities of daily living. It also imposes a heavy load on the family. According to the Global Burden of Disease 2019 (1), stroke ranks as the second cause of death and is the third leading cause of disability worldwide. It is estimated that 33–42% of patients continue to require assistance with activities of daily living 6 years after stroke and 36% remain disabled 5 years after stroke. Acute ischemic stroke accounts for 60–80% of all strokes (2). Intravenous thrombolysis (IVT) in the hyperacute phase (4.5 h) is an important measure for effective reperfusion of blood vessels and the promotion of neurological recovery. It has been recommended by domestic and international therapeutic guidelines as a first-level recommendation (3). But a considerable number of patients fail to benefit from thrombolysis, and even experience neurological deterioration (4). If the therapeutic effect of intravenous thrombolysis can be assessed and predicted as early as possible, the benefit of thrombolysis can be greatly improved. In this study, we investigated the risk factors affecting early thrombolytic efficacy in stroke patients. A nomogram was developed to help physicians make better decisions to maximize the benefit of thrombolysis.

## 2 Materials and methods

### 2.1 Study population

General data: 570 patients undergoing IVT treatment for ischemic stroke were enrolled between March 2018 and March 2022 from Dongyang People’s Hospital.

The inclusion criteria were as follows: (1) patients who were at least 18 years old; (2) patients who had been treated with rt-PA and onset time (referring to the time from symptom onset to thrombolytic treatment) ≤4.5 h; (3) patients who had a confirmed diagnosis of cerebral infarction with neurological deficits.

The exclusion criteria were as follows: (1) patients who had experienced hemorrhagic cerebral infarction; (2) patients who had undergone transient ischemic attack; (3) patients who had thrombosis of the cerebral venous sinus; (4) patients who had brain tumors; (5) patients who had incomplete primary observational indexes for diverse reasons; (6) patients who had contraindications to IVT; (7) coma, a pre-thrombolysis National Institutes of Health Stroke Scale (NIHSS) score of >25 points or epileptic seizure at the onset of illness; and (8) patients who had post-thrombolytic bridging for thrombolysis.

### 2.2 Data collection

This retrospective study collected baseline characteristics of patients, including general information (gender, age, alcohol consumption, smoking), a portion of previous medical history (diabetes mellitus, hypertension, atrial fibrillation, ischemic heart disease, stroke), history of previous medications (antiplatelet, statins), admission NIHSS score, pre-thrombolytic systolic blood pressure, pre-thrombolytic diastolic blood pressure, onset-to-treatment time (OTT), TOAST type, whether or not it was a posterior circulation infarction, antihypertensive drugs used before thrombolysis (HD) and laboratory findings (red blood cell count, hemoglobin, white blood cell count, blood platelet count, neutrophil count, lymphocyte count, monocyte count, lymphocyte to monocyte ratio (LMR), neutrophil to lymphocyte ratio (NLR), platelet to lymphocyte ratio (PLR), international normalized values (INR), prothrombin time (PT), activated partial thromboplastin time (APTT), fibrinogen (FIB), serum potassium, serum sodium, serum chloride, serum calcium, blood glucose level, blood urea nitrogen (BUN), creatinine). Stroke subtypes were determined by the investigators on the basis of an extended version of the TOAST (Trial of ORG10172 in Acute Stroke Treatment) classification. Arterial stenosis or occlusion was assessed using craniocervical CTA. Cerebral infarction caused by small artery occlusion type was defined as TOAST-A group according to the cerebral infarction etiology classification. Cerebral infarction due to large artery occlusion or stenosis, cardiogenic etiology, other etiologies, and unknown etiologies were defined as TOAST-B group. This study discussed the early thrombolytic efficacy after IVT. The National Institutes of Health Stroke Scale was used to measure early thrombolytic efficacy in stroke patients (5).

### 2.3 Definitions of outcomes

A decrease in the NIHSS score by <4 points with 24 h after thrombolysis was known as thrombolytic ineffective group. Meanwhile, a decrease in the NIHSS score by ≥4 points with 24 h after thrombolysis or 24 h NIHSS score ≤1 point was defined as thrombolytic effective group.

### 2.4 Statistical analysis

The respective expressions for continuous and categorical data were medians (quartiles) and number proportions. The statistical analyses employed to distinguish between the thrombolytically effective and thrombolytically ineffective groups were, as applicable, the unpaired *t*-test, Wilcoxon rank sum test, Pearson chi-square test, or Fisher’s exact test. Patients with cerebral infarction who underwent thrombolysis between March 2018 and March 2022 and met the inclusion criteria were randomly divided into a training group and a test group in an 8:2 ratio. The LASSO regression method and multivariate logistic regression were used to filter variables and choose predictors. Predictors were utilized to construct a predictive model for effective thrombolysis in cerebral infarction. The discriminatory ability of the model was determined by calculating the area under the curve (AUC). The clinical validity of the model was evaluated through decision curve analysis (DCA), while the calibration of the model was assessed using a calibration curve (6). The statistical software utilized was R 4.3.3. A significance level of *p* < 0.05 was applied to the results.

## 3 Results

**Figure 1:**
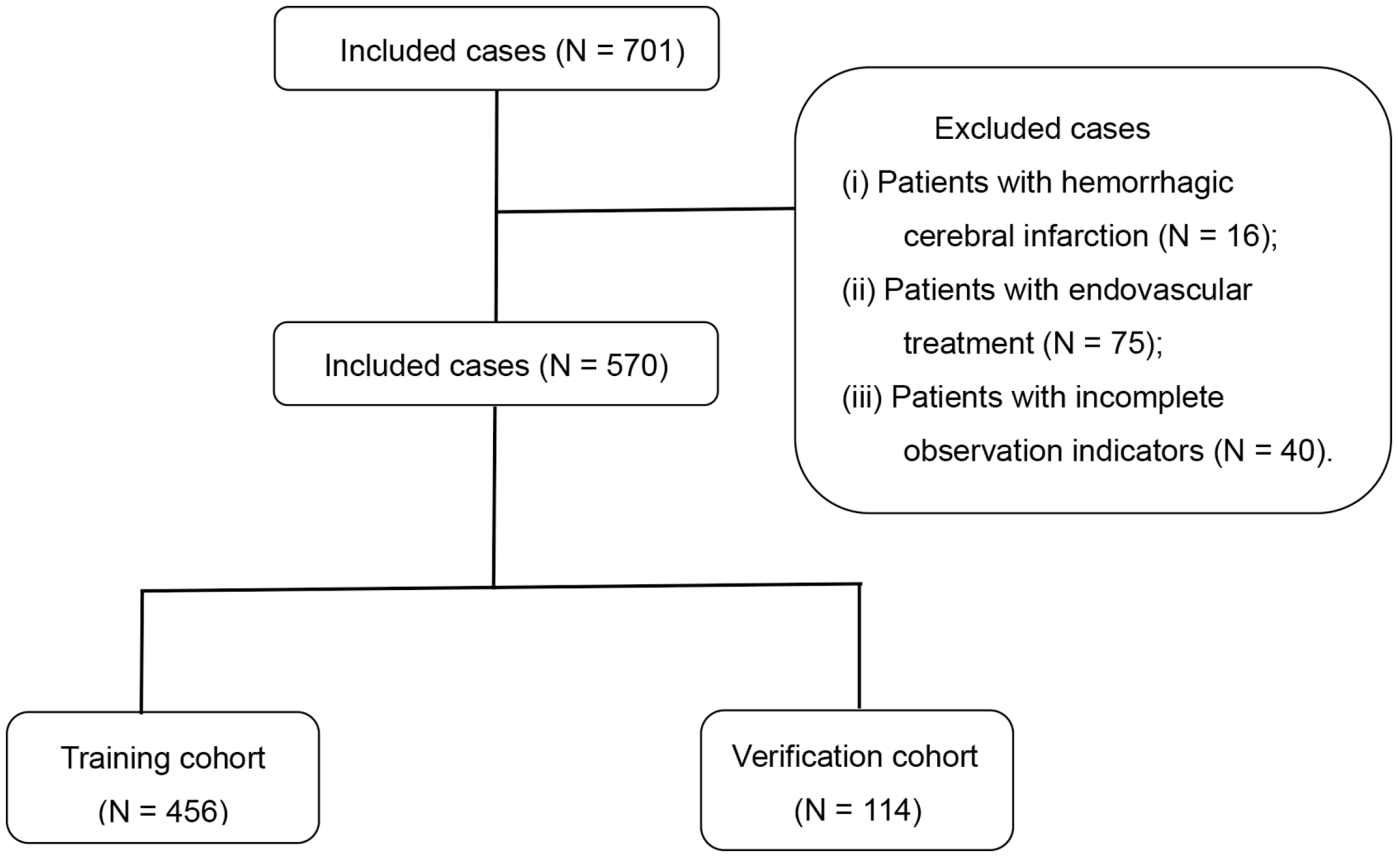
Flowchart of patient selection.

**Figure 2:**
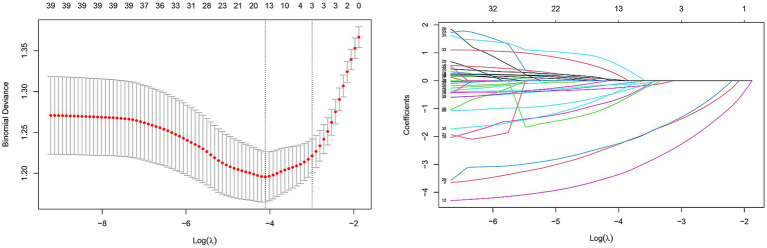
Predictor selection using the LASSO regression analysis with tenfold cross-validation. In this study, predictor’s selection was according to the 1-SE criteria (left dotted line), where 13 nonzero coefficients were selected. LASSO, least absolute shrinkage and selection operator; SE, standard error.

**Figure 3:**
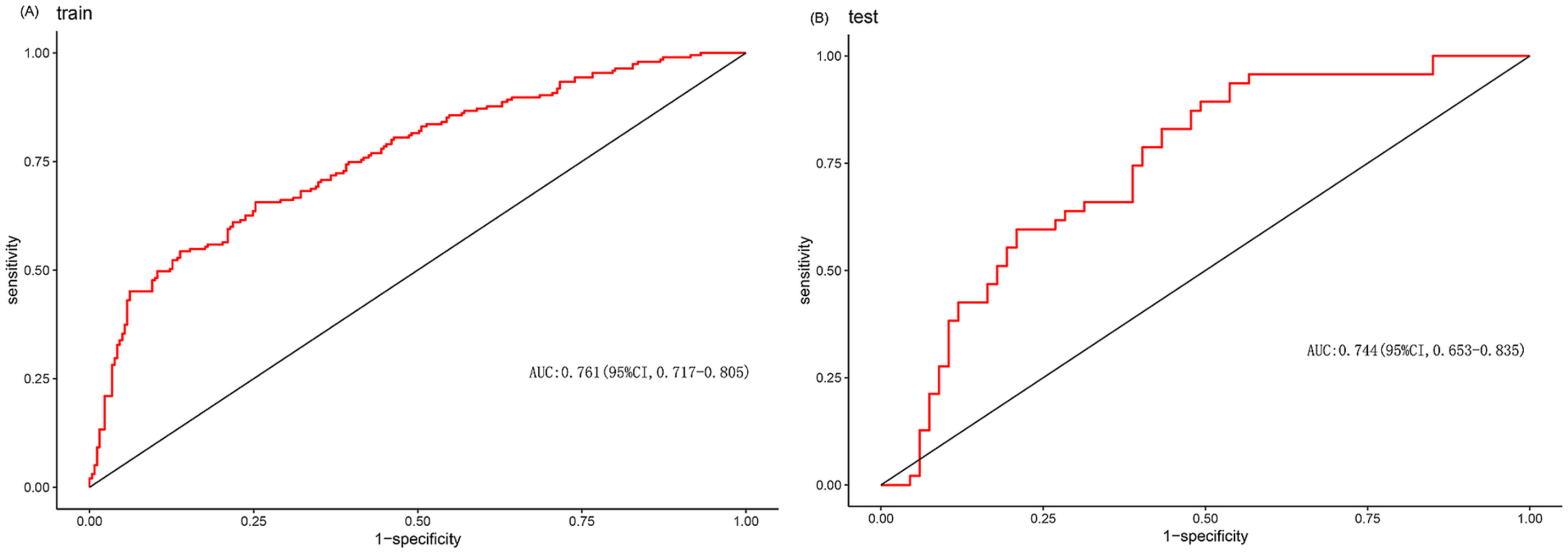
**(A)** ROC curve of the prediction model of train. **(B)** ROC curve of the prediction model of test.

**Figure 4:**
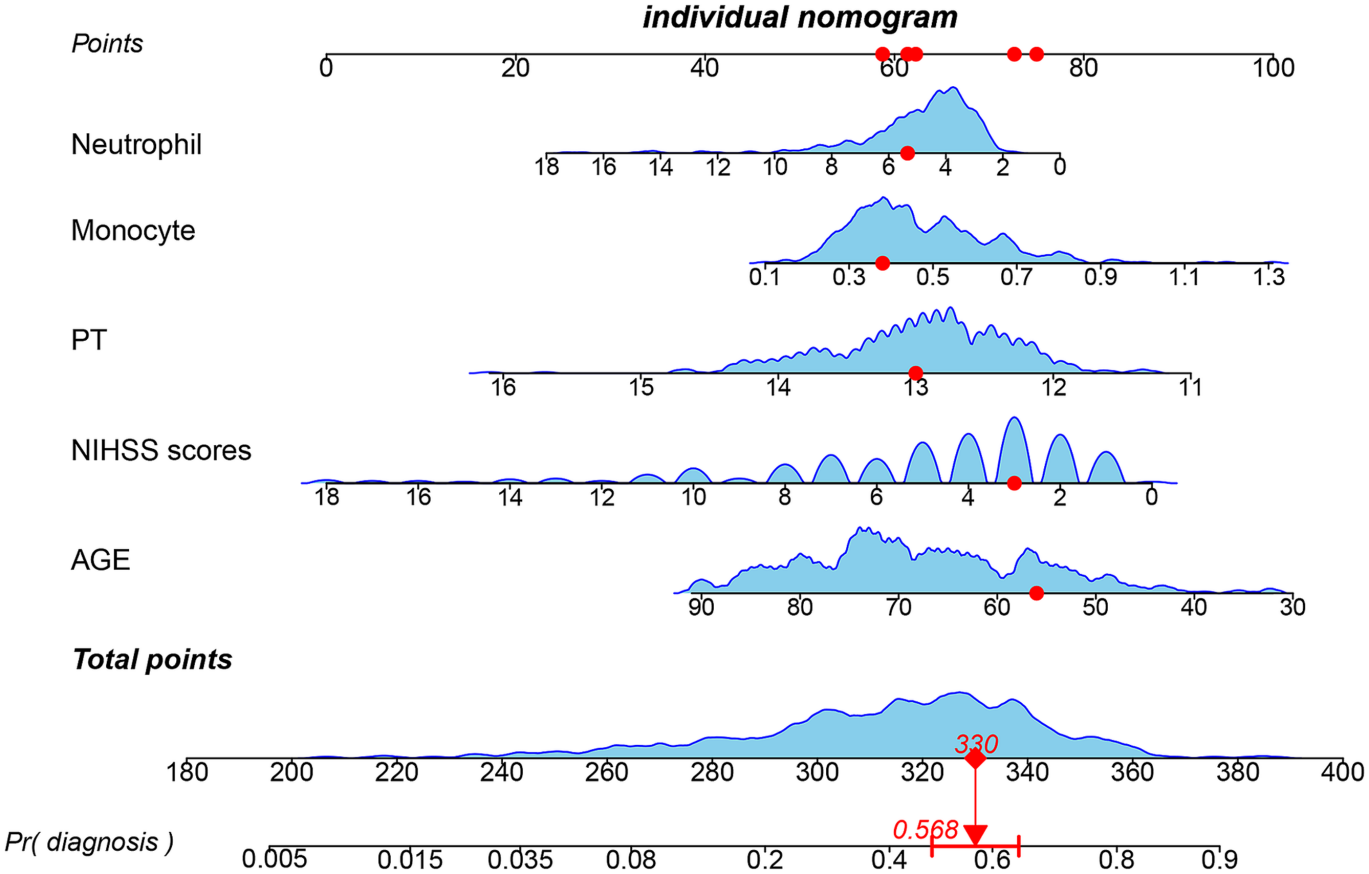
Nomogram for predicting early thrombolytic efficacy in stroke patients. The point of each independent predictor was the score corresponding to the upper scale, and the total points of each subject were the sum of the scores of each independent predictor.

**Figure 5:**
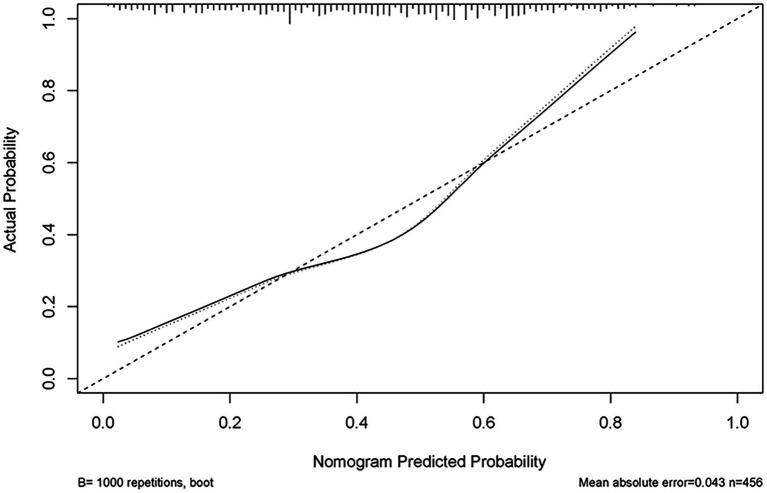
Calibration curve of the predictive model showing the degree of consistency between the predicted probability and observed probability.

**Figure 6:**
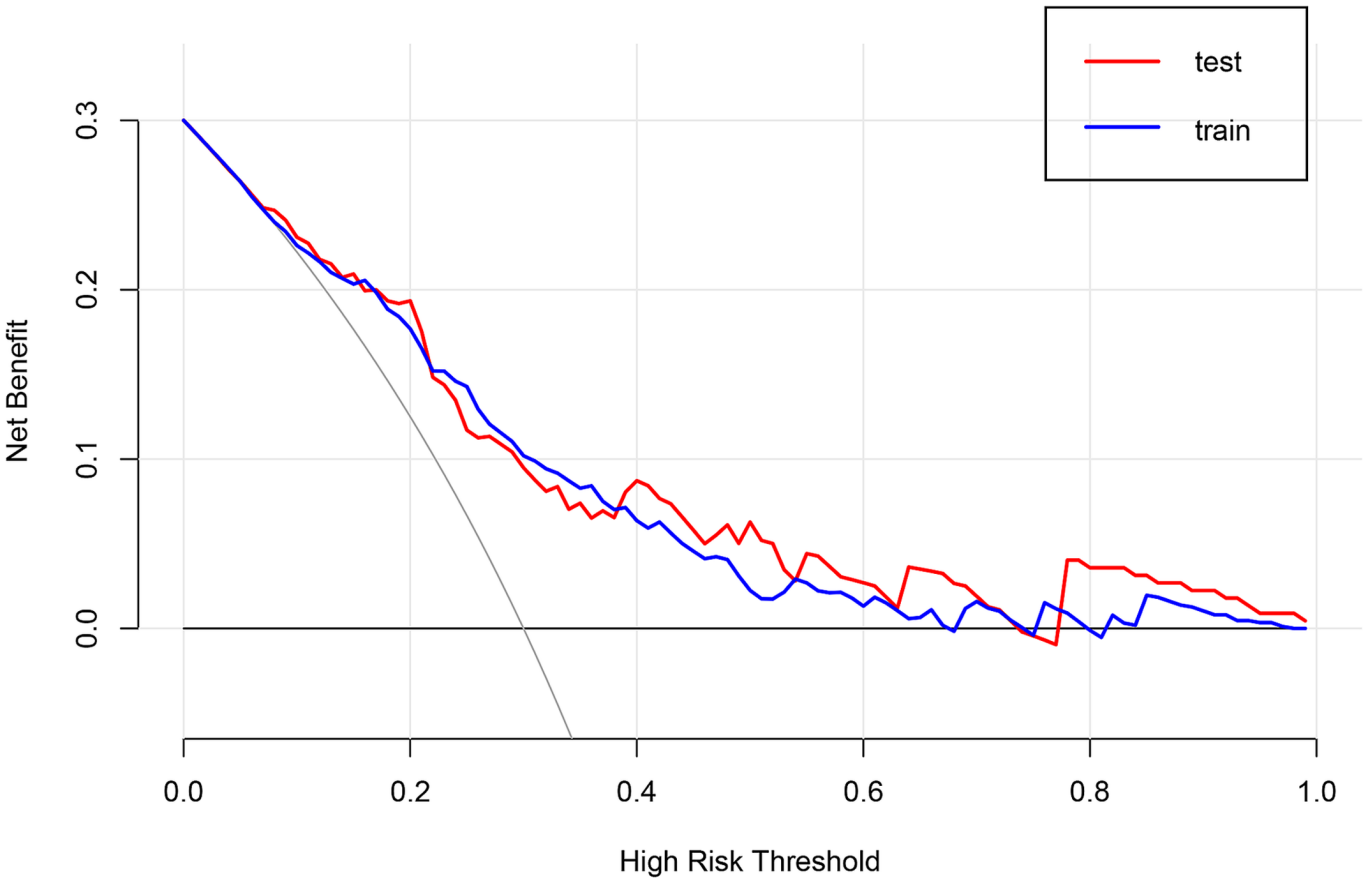
Decision curve analysis (DCA) of the nomogram.

We enrolled 701 stroke patients who received IVT treatment from Dongyang People’s Hospital from March 2018 and March 2022 and excluded patients with hemorrhagic cerebral infarction, endovascular treatment, incomplete observation indicators (Figure 1). Among the study participants, 42.5% (242/570) were patients with effective early thrombolytic therapy for cerebral infarction. The demographic and clinical characteristics of the study participants are presented (Table 1). A total of 13 variables were chosen from the 39 variables gathered from the patients based on non-zero coefficients calculated by LASSO regression analysis (Figure 2). These variables include age, history of diabetes, NIHSS score before thrombolysis, red blood cell count, neutrophil count, use of antiplatelet drugs, use of statins, monocyte count, serum potassium, serum calcium, APTT, OTT, and PT. In order to establish a predictive model for the early efficacy of thrombolytic therapy in ischemic stroke, a multivariate logistic regression analysis was conducted on the aforementioned 13 variables. Five statistically significant variables were selected, including age, NIHSS score before thrombolysis, PT, neutrophil count, and monocyte count (Table 2). The variance inflation factors for these five variables were also assessed, and no correlations were found (Table 3). The AUC of the prediction model on the training set was 0.761 (95% CI, 0.717–0.805), and the AUC on the internal validation set was 0.744 (95% CI, 0.653–0.835) (Figure 3). A nomogram was constructed to offer a practical and individualized instrument for forecasting the likelihood of early efficacy of thrombolysis in cerebral infarction (Figure 4). The calibration curve showed no statistical deviation between predicted and observed values (Figure 5). To assess its clinical application value, a decision curve analysis was additionally conducted. The decision curve showed that the threshold probabilities for the effectiveness of early thrombolysis in cerebral infarction were 25–67% and 25–73% in the training set and test set, respectively (Figure 6). The application of this nomogram to predict early thrombolytic efficacy in stroke patients could effectively assist clinicians in making better decisions. Let patients benefited more.

**Table 1:** Demographic and clinical characteristics of study participants.

Variables	Total (*n* = 570)	Ineffective group (*n* = 328)	Effective group (*n* = 242)	*p*-value
Gender, *n* (%)				0.478
Female	199 (35)	119 (36)	80 (33)	
Male	371 (65)	209 (64)	162 (67)	
Age (years), median (Q1, Q3)	69 (61, 76)	71 (63, 80)	65 (57, 73)	**<0.001**
Smoking (any cigarettes/week), *n* (%)				0.603
No	331 (58)	194 (59)	137 (57)	
Yes	239 (42)	134 (41)	105 (43)	
Alcohol use (≥ one unit/day), *n* (%)				1
No	336 (59)	193 (59)	143 (59)	
Yes	234 (41)	135 (41)	99 (41)	
Hypertension, *n* (%)				0.532
No	205 (36)	122 (37)	83 (34)	
Yes	365 (64)	206 (63)	159 (66)	
Diabetes mellitus, *n* (%)				0.084
No	468 (82)	261 (80)	207 (86)	
Yes	102 (18)	67 (20)	35 (14)	
Atrial fibrillation, *n* (%)				**0.012**
No	464 (81)	255 (78)	209 (86)	
Yes	106 (19)	73 (22)	33 (14)	
Coronary heart disease, *n* (%)				0.38
No	507 (89)	288 (88)	219 (90)	
Yes	63 (11)	40 (12)	23 (10)	
History of stroke, *n* (%)				0.69
No	492 (86)	281 (86)	211 (87)	
Yes	78 (14)	47 (14)	31 (13)	
Antiplatelet, *n* (%)				0.054
No	510 (89)	286 (87)	224 (93)	
Yes	60 (11)	42 (13)	18 (7)	
Statins, *n* (%)				0.08
No	517 (91)	291 (89)	226 (93)	
Yes	53 (9)	37 (11)	16 (7)	
NIHSS scores, median (Q1, Q3)	4 (3, 7)	5 (3, 7)	3 (2, 6)	**<0.001**
OTT (min), *n* (%)				0.051
0–180 min	442 (78)	263 (80)	179 (74)	
181–270 min	128 (22)	65 (20)	63 (26)	
TOAST, *n* (%)				**<0.001**
TOAST-A	328 (58)	169 (52)	159 (66)	
TOAST-B	242 (42)	159 (48)	83 (34)	
POCI, *n* (%)				0.688
No	425 (75)	242 (74)	183 (76)	
Yes	145 (25)	86 (26)	59 (24)	
HD, *n* (%)				0.878
No	473 (83)	271 (83)	202 (83)	
Yes	97 (17)	57 (17)	40 (17)	
SBP (mmHg), mean ± SD	151.19 ± 18.77	151.12 ± 18.73	151.29 ± 18.87	0.918
DBP (mmHg), median (Q1, Q3)	84 (74, 94)	84 (74, 94)	84 (74, 93)	0.668
Laboratory data, median (Q1, Q3)				
RBC (*10^12^/L)	4.61 (4.29, 4.95)	4.61 (4.28, 4.94)	4.61 (4.31, 4.97)	0.648
Hemoglobin (g/L)	144 (133, 154)	143 (132, 153)	145 (133, 155)	0.288
WBC (*10^9^/L)	7.22 (6.04, 8.84)	7.27 (6.08, 8.84)	7.17 (6.02, 8.81)	0.763
Neutrophil (*10^9^/L)	4.42 (3.57, 5.69)	4.46 (3.6, 5.72)	4.39 (3.48, 5.65)	0.332
Lymphocyte (*10^9^/L)	1.88 (1.44, 2.57)	1.87 (1.42, 2.51)	1.88 (1.47, 2.74)	0.156
Monocyte (*10^9^/L)	0.43 (0.35, 0.56)	0.43 (0.34, 0.54)	0.43 (0.36, 0.58)	0.197
NLR	2.28 (1.55, 3.32)	2.33 (1.72, 3.41)	2.15 (1.49, 3.24)	0.052
PLR	106.69 (78.91, 146.42)	107.53 (80.21, 151.87)	105.5 (77.33, 139.5)	0.534
LMR	4.37 (3.27, 5.82)	4.36 (3.26, 5.66)	4.49 (3.27, 5.93)	0.453
Platelet (*10^9^/L)	207 (169.25, 244.5)	205 (167.75, 240.25)	211 (173.25, 250.5)	0.056
PT(s)	12.9 (12.5, 13.4)	13.1 (12.7, 13.6)	12.8 (12.4, 13.1)	**<0.001**
APTT(s)	34 (31.63, 36.8)	34.1 (31.6, 37.02)	33.8 (31.72, 36.5)	0.25
INR	0.99 (0.95, 1.04)	1 (0.96, 1.05)	0.98 (0.94, 1.01)	**<0.001**
Fibrinogen (g/L)	3.22 (2.78, 3.75)	3.22 (2.81, 3.72)	3.24 (2.73, 3.79)	0.567
Potassium (mmol/L)	3.8 (3.56, 4.05)	3.79 (3.55, 4.09)	3.82 (3.57, 4.04)	0.907
Sodium (mmol/L)	139.3 (137.4, 141)	139.1 (137.4, 141)	139.5 (137.52, 141)	0.633
Chlorinum (mmol/L)	102.7 (100.03, 105)	102.7 (100.18, 104.93)	102.7 (99.9, 105.18)	0.896
Calcium (mmol/L)	2.29 (2.24, 2.36)	2.29 (2.24, 2.37)	2.29 (2.24, 2.36)	0.559
Blood glucose level (mmol/L)	6.92 (6.01, 8.54)	7.16 (6.03, 9.07)	6.78 (5.98, 8.19)	0.087
BUN (mmol/L)	5.6 (4.7, 7.07)	5.6 (4.68, 7.1)	5.75 (4.7, 6.9)	0.945
Serum creatinine (μmol/L)	72 (60, 84)	71 (59, 84)	73 (61.25, 84)	0.511

Data are presented as mean ± standard deviation or *N* (%) or median (interquartile range). TOAST-A, small-artery occlusion lacunar; TOAST-B, large-artery atherosclerosis, cardio embolism, acute stroke of other determined etiology or stroke of other undetermined etiology; POCI, post circulation infarction; HD, antihypertensive drugs used before thrombolysis; NLR, neutrophil-lymphocyte ratio; PLR, platelet-lymphocyte ratio; LMR, lymphocyte-monocyte ratio; OTT, onset-to-treatment time; NIHSS, National Institutes of Health Stroke Scale; SBP, systolic blood pressure; DBP, diastolic blood pressure; WBC, white blood cell; RBC, red blood cell; PT, prothrombin time; APTT, active partial thromboplastin; INR, international normalized ratio; BUN, blood urea nitrogen.

Bold values indicate a significant difference between the ineffective group and effective group (*p* < 0.05).

**Table 2:** The multiple logistic regression analysis of risk factors for predicting early thrombolytic efficacy in the training group.

Variables	Logistic regression	
	OR (95% CI)	*p*
Age	0.958 (0.939–0.976)	<0.001
NIHSS scores	0.818 (0.756–0.881)	<0.001
PT	0.548 (0.382–0.775)	<0.001
Neutrophil	0.883 (0.785–0.984)	0.008
Monocyte	6.270 (1.640–25.30)	0.030

**Table 3:** Collinearity of combinations of variables in the training group.

Variable	Variation inflation factor
Age	1.034
NIHSS scores	1.011
PT	1.031
Neutrophil	1.141
Monocyte	1.140

## 4 Discussion

There are several effective treatment options for patients with acute ischemic stroke. Among these, IVT and endovascular intervention are the most important treatment methods. Suitable patients can combine the two (7, 8). However, IVT remains the preferred option for a considerable proportion of patients. In this study, we retrospectively analyzed the early thrombolytic outcome of 570 stroke patients, with the expectation of identifying factors affecting the efficacy of thrombolysis. It can help acute neurologists to make an immediate assessment of the patient. Let patients benefit from thrombolysis to the greatest extent possible. This study showed that age, NIHSS score before thrombolysis, prothrombin time, neutrophil count, and monocyte count were predictive factors affecting the early efficacy of thrombolysis in cerebral infarction.

Previous studies have shown that old age was one of the most significant and independent predictors of death and poor prognosis in stroke (9, 10). Shi’s et al. (11) study also showed that older patients with acute cerebral infarction have a higher risk of neurological deterioration after revascularization, especially in subgroup analyses, where the symptoms were more pronounced in patients who received IVT treatment. Age-related neural function deterioration may be mechanistically linked to two factors: elevated brain levels of phosphorylated adenosine monophosphate-activated protein kinase (pAMPK) and reduced Na-K-Cl cotransporter expression (12, 13). However, the specific mechanism requires further investigation.

In addition, the NIHSS score is also independent factors in influencing early thrombolytic efficacy in stroke patients. The severity of a patient’s condition is positively correlated with its NIHSS score. Therefore, a substantial number of thrombolysis prognostic models have used the NIHSS score on admission as a variable (14, 15). Adams et al. (16) concluded that baseline NIHSS score was strongly associated with prognosis. A pre-thrombolysis NIHSS score of ≥16 was associated with death and dependence, whereas a pre-thrombolysis NIHSS score of ≤6 suggested a good prognosis. Pre-thrombolysis NIHSS score is a significant predictor of thrombolytic efficacy. A higher NIHSS score often indicates a larger area of cerebral infarction and malignant cerebral edema. Although our study found that patients with small artery cerebral infarction often have better early thrombolytic efficacy than patients with other types of cerebral infarction, multivariate regression analysis showed that stroke subtype should not be a primary consideration. At the same time, the early efficacy of thrombolysis in cerebral infarction is unrelated to whether it is an anterior or posterior circulation cerebral infarction.

In patients with acute cerebral infarction, intravenous thrombolytic therapy dissolves thrombi by activating the fibrinolytic system. The pathological process is accompanied by the interaction between the coagulation and fibrinolytic systems. PT refers to the time it takes for blood to clot after adding thrombin and calcium to platelet-poor plasma, reflecting the efficiency of the extrinsic coagulation pathway initiated by tissue factor. Zhao et al. (17) found that the level of baseline PT after rt-PA thrombolysis was significantly associated with the 3-month unfavorable functional outcome in stroke patients, but no association was found between PT and early neurological deterioration (END). In this study, we found that the baseline PT before thrombolysis was an important independent factor influencing the early efficacy of thrombolysis in cerebral infarction. A shorter PT was associated with better early therapeutic outcomes in thrombolysis for cerebral infarction.

Neuroinflammatory responses also play an important role in the pathophysiology of ischemic stroke. Inflammatory cytokines or chemokines are immediately formed after cerebral ischemia, stimulating the expression of adhesion molecules on leukocytes and endothelial cells, causing various inflammatory cells (neutrophils, monocytes/macrophages, different subtypes of T cells, and other inflammatory cells) to enter the ischemic area, exacerbating brain damage. Neutrophils induce the expression of matrix metalloproteinase-9 (MMP-9) by producing proinflammatory cytokines, which leads to the destruction of the blood–brain barrier and further brain damage (18, 19). Our study found that baseline neutrophil count and monocyte count before thrombolysis may be independent predictors of early thrombolytic efficacy in cerebral infarction. NLR, PLR, and LMR are novel inflammatory prediction indicators. Sun et al. (20) found that a high PLR value 24 h after rt-PA treatment was independently associated with poor prognosis and increased risk of death. However, the PLR value at admission was not significantly associated with poor prognosis and increased risk of death. Multiple studies have shown that the combination of NLR and low LMR observed within 24 h after thrombolysis can serve as an independent predictor of poor outcomes at 3 months in acute cerebral infarction patients (21, 22). Changes in NLR, PLR, and LMR after hospitalization have not been included in this study. No association was found between baseline NLR, PLR, and LMR and early efficacy after thrombolysis for cerebral infarction.

Time is the brain. The length of the therapeutic time may also influence the efficacy of thrombolysis in cerebral infarction. OTT have been proven to be an independent predictor of END following thrombolysis in cerebral infarction by multiple studies (11, 23). Although intravenous thrombolysis is generally considered effective within 3–4.5 h after onset, the possibility of END also increases within this window (24). However, in our study, we did not find that patients who underwent thrombolysis for cerebral infarction within 0–3 h had better early neurological improvement than those who underwent thrombolysis for cerebral infarction within 3–4.5 h.

This study has several limitations. First, it was a retrospective study, and selection bias was unavoidable. Second, it was a single-center study, and external validation might be needed to support our conclusions. Third, this study only examined the early efficacy of thrombolysis in cerebral infarction and did not examine the condition of patients three months after thrombolysis, which might bias the assessment of long-term prognosis.

## 5 Conclusion

A prediction model for early thrombolytic efficacy in stroke patients was developed in this study. These predictive factors are relatively easy to obtain in clinical practice. The nomogram can help clinicians make better clinical decisions in acute cerebral infarction thrombolysis. Let more patients benefit from thrombolysis.

## Data Availability

The original contributions presented in the study are included in the article/supplementary material, further inquiries can be directed to the corresponding author.
